# Donor-Related Determinants of Platelet Yield and Adverse Reactions in Plateletpheresis Procedures in a Tertiary Care Cancer Center in South India

**DOI:** 10.7759/cureus.101711

**Published:** 2026-01-16

**Authors:** Lekshmi Sudev, Vijayalakshmi K

**Affiliations:** 1 Transfusion Medicine, Regional Cancer Centre, Thiruvananthapuram, IND

**Keywords:** adverse donor reactions, apheresis donor safety, body mass index, donor characteristics, high-yield plateletpheresis, plateletpheresis, platelet yield, pre-donation platelet count, single-donor platelets, transfusion medicine

## Abstract

Background

Plateletpheresis is increasingly central to modern transfusion practice, with rising demand for high-yield single-donor apheresis platelets. Optimizing platelet yield while preserving donor safety is critical, particularly as services adopt higher target-yield strategies. This study evaluated donor-level determinants of platelet yield and adverse donor reactions and compared donor safety across different target-yield categories in routine practice.

Methodology

In this prospective, observational study, 670 plateletpheresis procedures performed at a tertiary cancer center between January and June 2025 were analyzed. Platelet yield (×10¹¹ platelets) and occurrence of any donor adverse reaction (yes/no) were defined as the primary outcome variables. Donor age, body mass index (BMI), hemoglobin concentration, hematocrit, and pre-donation platelet count were evaluated as predictor variables. Associations between donor characteristics and platelet yield were assessed using Pearson correlation and multivariable linear regression. Associations between donor characteristics and adverse reactions were analyzed using multivariable logistic regression. Adverse reaction rates were compared across predefined target-yield categories (3.0, 4.5, and 6.0 × 10¹¹ platelets) using the chi-square test.

Results

The mean platelet yield was 4.4 ± 1.4 × 10¹¹ platelets. Pre-donation platelet count showed the strongest correlation with yield (r = 0.758, p < 0.001) and emerged as the dominant independent predictor in multivariable analysis (standardized β = 0.657). BMI also independently predicted yield (β = 0.265), while hematocrit demonstrated a modest but statistically significant inverse association. Adverse donor reactions occurred in 10.1% of procedures, predominantly mild. Higher BMI was independently associated with a lower risk of adverse reactions (odds ratio = 0.93 per kg/m²; 95% confidence interval = 0.87-0.99). No significant difference in adverse reaction rates was observed across target-yield categories (p = 0.785).

Conclusions

Pre-donation platelet count was the strongest determinant of plateletpheresis yield. BMI contributed modestly to higher yields and was associated with a lower likelihood of donor adverse reactions, while hematocrit showed a small inverse association with yield. Donor reactions were infrequent and predominantly mild. Higher target-yield plateletpheresis was not associated with an increased risk of adverse reactions within the applied donor selection framework. These findings suggest that higher-yield plateletpheresis can be implemented in appropriately selected donors without an observed compromise in donor safety.

## Introduction

Platelet transfusion is an essential component of supportive care in hematologic malignancies, intensive chemotherapy, and major surgical interventions. The therapeutic effectiveness of platelet transfusion is closely linked to the dose and quality of the platelet component, with higher platelet yields producing superior post-transfusion increments and reducing the frequency of transfusions required to maintain hemostasis [1,2]. This recognition has driven a marked global shift toward single-donor apheresis platelets (SDPs), which offer several advantages over pooled random-donor platelets, including reduced donor exposure, improved product consistency, and lower risks of transfusion-transmitted infections, alloimmunization, and febrile reactions [3].

As clinical demand increased, transfusion services have increasingly adopted high-yield and double-dose plateletpheresis, supported by advances in apheresis technologies that allow greater volumes of donor blood to be processed efficiently and safely. As utilization has expanded, so too has the need for a reliable pool of eligible apheresis donors from whom high-quality, high-yield products can be consistently obtained to maximise post-transfusion platelet recovery. A critical determinant of product adequacy is platelet yield, which varies widely across donors. Donor factors implicated in determining platelet yield include demographic, anthropometric, and hematological variables, such as age, sex, body size, hemoglobin concentration, total leukocyte count, hematocrit, platelet count, and platelet indices, with pre-donation platelet count consistently identified as the most significant determinant [4-6].

The increasing adoption of higher target-yield categories (e.g., 4.5 × 10¹¹ and ≥6 × 10¹¹) also brings renewed attention to donor safety [7,8]. Although contemporary apheresis systems are generally well tolerated, donor adverse reactions, primarily hematoma, citrate-related symptoms, and phlebotomy complications, remain clinically relevant. Reaction rates in published Indian studies range from approximately 4% to 18%, with most events being mild and self-limiting [9-11]. Notably, high-yield procedures do not appear to confer a significantly greater overall risk compared with standard-yield collections [8,12], but some subgroups (e.g., first-time donors, younger or lower body mass index (BMI) donors) may be more susceptible to vasovagal or citrate-related events [13-15]. Understanding which donor characteristics influence adverse reaction profiles is critical for maintaining donor wellbeing, refining eligibility criteria, and sustaining voluntary donor pools.

Platelet yield optimization and donor safety are intrinsically linked in routine plateletpheresis practice, yet most published studies, particularly from single-center Indian settings, have examined yield determinants and donor adverse reactions as separate outcomes. In real-world transfusion services, donors are not randomly assigned to different target-yield protocols; rather, higher target yields are selectively pursued in donors with favorable baseline characteristics such as higher platelet counts, larger body size, and greater estimated blood volume. These same characteristics also influence the likelihood of donation-related adverse reactions. Consequently, studies that analyze yield and donor safety independently fail to capture the practical decision-making context faced by transfusion services, where both outcomes must be balanced simultaneously within the same donor population. An integrated evaluation of donor predictors, platelet yield, and adverse reactions within a single cohort therefore addresses a critical knowledge gap by reflecting routine practice and generating evidence that directly informs donor selection and target-yield decisions in plateletpheresis services.

Therefore, this study aims to evaluate the impact of donor characteristics on platelet yield, assess the influence of donor variables on the occurrence of adverse reactions, and determine whether adverse reaction rates differ across target-yield groups in routine practice.

## Materials and methods

Study design and setting

This prospective, observational study included 670 plateletpheresis SDP procedures conducted in the Division of Transfusion Medicine, Regional Cancer Centre, Trivandrum, over a period of six months from January 1, 2025, to June 30, 2025. Ethical approval for the study was obtained from the Human Ethics Committee, Regional Cancer Centre, Thiruvananthapuram (HEC No. 29/25), and the study was conducted in accordance with national ethical and Good Clinical Practice guidelines. Written informed consent was obtained from all participants.

Donor selection

Donors were required to meet the following criteria: age within the accepted donation range, male sex, body weight ≥55 kg, hemoglobin ≥12.5 g/dL, total leukocyte count within normal limits, and a pre-donation platelet count ≥150 × 10⁹/L. Eligibility for double-unit plateletpheresis required a donor body weight of ≥60 kg and a pre-donation platelet count ≥250 × 10⁹/L. Eligibility also required compliance with the Guidelines for Blood Donor Selection and Blood Donor Referral by the National Blood Transfusion Council [16]. Additional criteria included written informed consent, suitable veins in both cubital fossae, achievement of a minimum target yield of 3.0, 4.5, or 6.0 × 10¹¹ platelets, as applicable, and availability of post-donation samples. Donors were excluded if they were repeat donors, screened reactive for transfusion-transmitted infections (hepatitis B, hepatitis C, HIV, syphilis, or malaria), or if their hematological indices were abnormal. Malaria was tested using a rapid card assay, while the remaining infections were assessed by chemiluminescence immunoassay.

Plateletpheresis procedure and sample analysis

For each donor, demographic details and baseline hematological parameters were recorded. Plateletpheresis was performed using the Trima Accel® cell separator with single-needle, closed-system apheresis kits, maintaining a blood-to-anticoagulant ratio between 9:1 and 11:1. All collected platelet units were leukoreduced, with residual leukocyte counts of <1 × 10⁶ white blood cells per unit. The device software was initialized with donor sex, weight, height, and counts, and the system calculated the blood volume required to achieve the minimum target yield. Peripheral blood samples were collected in EDTA tubes: 5 mL pre-procedure for complete blood counts, grouping, and infection screening, and 2 mL post-procedure for platelet count estimation. Counts were performed on an automated hematology analyzer (Automatic Medonic Hematology Analyzer, M32). For this, 2-mL product samples were aseptically withdrawn after gentle mixing. Products were stored in standard SDP bags on a platelet agitator until day-one testing. Plateletpheresis procedures that were terminated prematurely due to an adverse event before achievement of the predefined target platelet yield were excluded from the analysis.

Donor adverse reactions were monitored throughout the plateletpheresis procedure and for 30 minutes post-donation, in accordance with institutional standard operating procedures. Adverse reactions were recorded based on clinical observation and donor-reported symptoms and categorized by type, including hematoma, citrate-related symptoms, and other minor reactions such as giddiness or nausea/vomiting. Formal severity grading was not performed, as the majority of reactions were mild and self-limiting.

Variables

The following donor, procedural, and product-related parameters were recorded: donor age, sex, weight, height, hemoglobin concentration, hematocrit (%), pre- and post-donation platelet counts, total blood volume processed, procedure duration, volume of acid citrate dextrose-A used, product platelet count, product volume, and donor reactions.

BMI was calculated from the height and weight recorded for each donor using the following formula:

\[ BMI = Weight\ (kg) / Height\ (m^2) \]

Platelet yield was calculated as follows:

\[ Yield = Product\ volume\ (mL) \times Platelet\ count\ per\ \mu L \times Conversion\ factor \]

Statistical analysis

Continuous variables were summarized as mean with standard deviation (SD) or with other descriptive statistics (median, interquartile range (IQR), minimum, maximum) as appropriate. Categorical variables were expressed as counts and percentages. The association between donor characteristics (age, BMI, pre-donation platelet count, hemoglobin concentration, and hematocrit) and platelet yield was initially explored using Pearson correlation analysis. To identify independent predictors of platelet yield, a multiple linear regression model was constructed with platelet yield as the dependent variable and the above donor characteristics as independent predictors. For safety analysis, the occurrence of any adverse donor reaction (yes/no) was defined as the outcome variable. As adverse reactions were categorical in nature, correlation analysis was not performed. Instead, associations between donor characteristics (age, BMI, pre-donation platelet count, hemoglobin, and hematocrit) and adverse reactions were evaluated using multivariable logistic regression, and results were expressed as odds ratios (ORs) with 95% confidence intervals (CIs). Differences in the proportion of donors experiencing any adverse reaction versus none across predefined target-yield categories (3.0, 4.5, and 6.0 × 10¹¹ platelets) were assessed using the Pearson chi-square test. All statistical tests were two-tailed, and a p-value <0.05 was considered statistically significant. All analyses were performed using R software (version 4.5.1, R Foundation for Statistical Computing, Vienna, Austria).

## Results

A total of 670 plateletpheresis donors were included in the analysis. The baseline demographic and hematological characteristics are summarized in Table 1. The cohort comprised predominantly young to middle-aged adults, with a median age of 33 years (IQR = 28-39). The donors demonstrated a healthy hematologic profile, with a median hemoglobin level of 15.5 g/dL (IQR = 14.8-16.1) and a corresponding median hematocrit of 44% (IQR = 42.0-45.4). Pre-donation platelet counts ranged from 156-376 × 10³/µL, with a median of 250 × 10³/µL.

**Table 1:** Baseline demographic and hematological characteristics of platelet donors (N = 670). Note: Values are presented as minimum–maximum, median with interquartile range (IQR), and mean ± standard deviation (SD), as appropriate. Platelet yield is reported in ×10¹¹ platelets and pre-donation platelet count in ×10³/µL.

Parameter	Minimum–Maximum	Median	IQR	Mean ± SD
Age (in years)	18.0–55.0	33.0	28.0–39.0	33.8 ± 8.2
Body mass index (kg/m²)	19.0–34.0	27.0	24.0–30.6	27.3 ± 4.2
Pre-donation platelet count (×10³/µL)	156.0–376.0	250.0	221.0–278.8	250.8 ± 43.8
Hemoglobin (g/dL)	12.6–18.5	15.5	14.8–16.1	15.5 ± 1.0
Hematocrit (%)	36.9–51.2	44.0	42.0–45.4	43.9 ± 2.7

**Figure 1:**
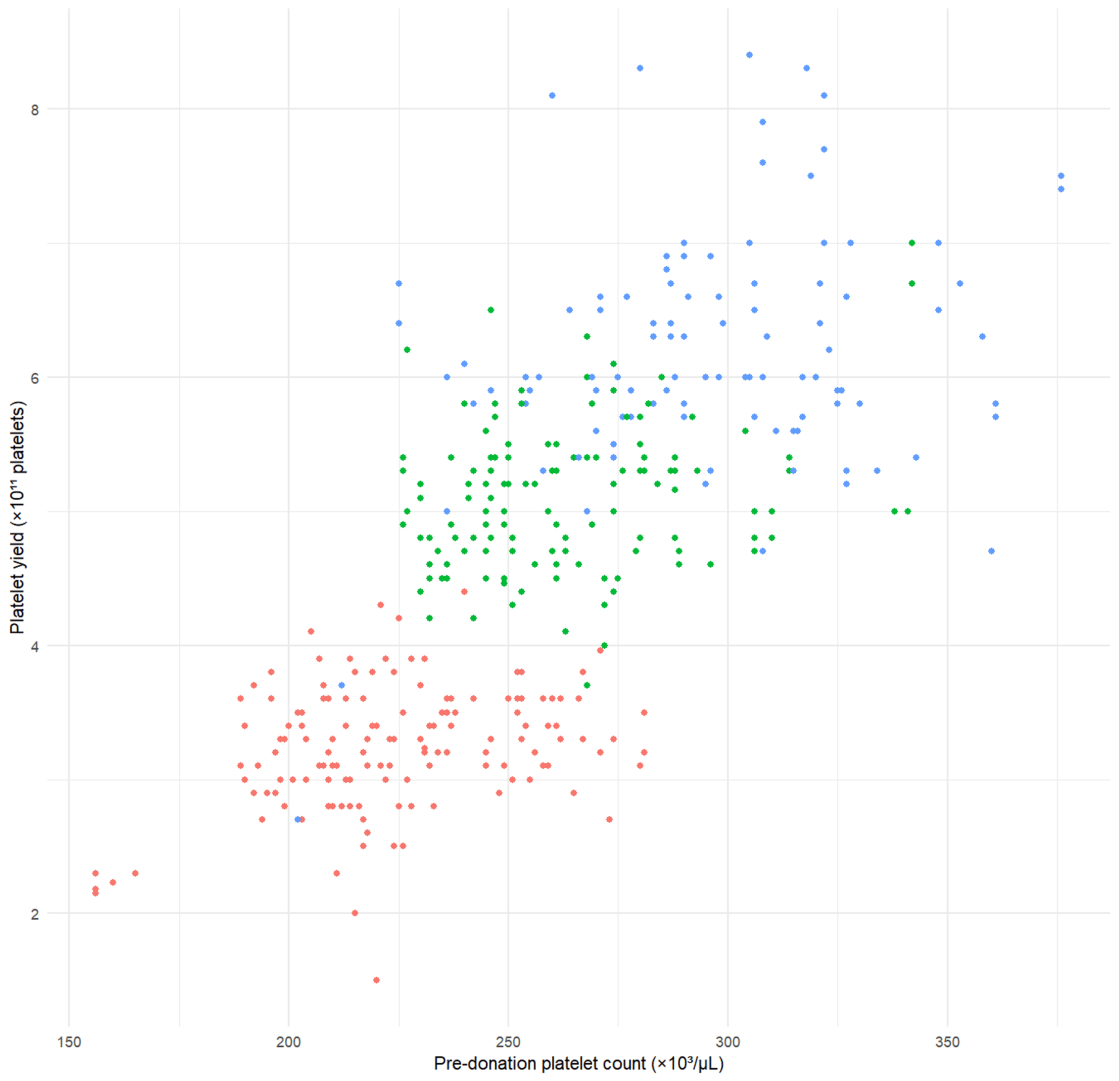
Relationship between pre-donation platelet count and platelet yield. Scatter plot depicting the relationship between pre-donation platelet count and platelet yield in single-donor plateletpheresis. Each point represents an individual donor. Points are color-coded according to target platelet yield categories: 3 × 10¹¹ platelets (red), 4.5 × 10¹¹ platelets (green), and 6 × 10¹¹ platelets (blue).

The mean platelet yield achieved was 4.4 ± 1.4 × 10¹¹ platelets. Table 2 presents the Pearson correlation between donor variables and platelet yield. Pre-donation platelet count showed the strongest positive correlation with yield (r = 0.758, p < 0.001), indicating that higher baseline platelet counts were closely linked to greater procedural yield. This relationship is visually reinforced in Figure 1, where platelet yield rises progressively with increasing pre-donation platelet count, and distinct clustering across target-yield categories (3.0, 4.5, and 6.0 × 10¹¹ platelets) further demonstrates the strong upward gradient. Donors with higher baseline counts consistently achieved higher yields, regardless of the target setting. BMI also demonstrated a moderate positive correlation (r = 0.520, p < 0.001). Age exhibited a weak but statistically significant negative correlation with platelet yield (r = -0.113, p = 0.003), suggesting a small decline in yield with increasing donor age. Hemoglobin level showed a non-significant negative association (r = -0.072, p = 0.063), while hematocrit displayed a significant inverse relationship (r = -0.105, p = 0.007). Collectively, these findings highlight that both baseline platelet reserve and donor body habitus are influential determinants of yield, with the dominance of platelet count clearly illustrated both statistically and graphically.

**Table 2:** Correlation of donor variables with platelet yield (N = 670). Note: r = Pearson correlation coefficient. P-values reflect the significance of the linear association between each variable and platelet yield. Statistical significance codes: * p < 0.05, ** p < 0.01, *** p < 0.001. Pre-donation platelet count is expressed as ×10³/µL; platelet yield is expressed as ×10¹¹ platelets.

Parameter	r (correlation with platelet yield)	P-value
Age (in years)	-0.113	0.003^**^
Body mass index (kg/m²)	0.520	<0.001^***^
Pre-donation platelet count (×10³/µL)	0.758	<0.001^***^
Hemoglobin (g/dL)	-0.072	0.063
Hematocrit (%)	-0.105	0.007^**^

Table 3 presents the results of the multiple linear regression analysis assessing the independent predictors of platelet yield. Column 2 shows the non-standardized coefficients, which indicate the absolute change in yield (×10¹¹ platelets) for each unit change in the predictor. Column 3 displays the standardized coefficients, allowing comparison of the relative strength of each variable within the model. The standardized coefficients demonstrate that pre-donation platelet count (β = 0.657) was the strongest independent predictor of platelet yield, followed by BMI (β = 0.265), which also contributed meaningfully. Hematocrit showed a small negative standardized effect (β = -0.147), while age and hemoglobin had standardized values close to zero, indicating minimal independent influence after adjustment. Compared with all other predictors, pre-donation platelet count showed the largest effect size, confirming its dominant role in determining final platelet yield.

**Table 3:** Multiple linear regression analysis of donor variables predicting platelet yield (N = 670). Note: Regression coefficients represent the adjusted association between each donor variable and platelet yield (×10¹¹ platelets). Standardized coefficients (β) indicate the relative strength of each predictor. Statistical significance codes: p < 0.05*, p < 0.01**, p < 0.001***.

Predictor	Coefficient	Standard coefficient	P-value
Age (in years)	-0.001	-0.003	0.887
Body mass index (kg/m²)	0.089	0.265	<0.001^***^
Pre-donation platelet count (×10³/µL)	0.021	0.657	<0.001^***^
Hemoglobin (g/dL)	0.115	0.080	0.133
Hematocrit (%)	-0.077	-0.147	0.006^**^

Adverse donor reactions occurred in 10.1% of plateletpheresis procedures. Hematoma (4.5%) and citrate toxicity (3.1%) accounted for the majority of events, whereas other reactions, such as giddiness and nausea or vomiting, were relatively uncommon (2.5%). In multivariable logistic regression analysis, higher donor BMI was independently associated with a significantly lower likelihood of adverse reactions (OR = 0.93 per kg/m²; 95% CI = 0.87-0.99; p = 0.035) (Table 4). No significant independent associations were observed for age, pre-donation platelet count, hemoglobin, or hematocrit.

**Table 4:** Multivariable logistic regression analysis of donor characteristics associated with adverse donor reactions (N = 670). Note: Odds ratios (ORs) and 95% confidence intervals (CIs) were estimated using multivariable logistic regression. The dependent variable was the occurrence of any adverse donor reaction (yes/no). An OR >1 indicates increased odds of an adverse reaction, whereas an OR <1 indicates reduced odds. Statistical significance codes: p < 0.05*, p < 0.01**, p < 0.001***.

Predictor	OR	95% CI	P-value
Age (in years)	1.02	0.99, 1.05	0.156
Body mass index (kg/m²)	0.93	0.87, 0.99	0.035^*^
Pre-donation platelet count (×10³/µL)	1.00	1.00, 1.01	0.163
Hemoglobin (g/dL)	1.31	0.74, 2.15	0.312
Hematocrit (%)	0.95	0.79, 1.17	0.628

Because the study utilized preset target-yield thresholds (3.0, 4.5, and 6.0 × 10¹¹) in the collection process, the distribution of adverse reactions was compared across these categories as an additional safety assessment. Table 5 presents the types of adverse reactions observed across the three target-yield categories. Adverse donor reactions were infrequent across all target-yield categories. Hematoma and citrate toxicity each occurred in ≤4% of procedures across all categories, while other adverse reactions, including nausea, vomiting, and giddiness, were reported in 2.5-6.5% of cases.

**Table 5:** Types of adverse reactions observed in the study population (N = 670). Note: Frequencies and percentages are calculated within each target-yield category: 3.0 × 10¹¹ platelets (n = 312), 4.5 × 10¹¹ platelets (n = 200), and 6.0 × 10¹¹ platelets (n = 158). The Other category comprises distinct adverse reactions, including nausea, vomiting, and giddiness.

Adverse reactions	Target yield					
	3 × 10¹¹		4.5 × 10¹¹		6 × 10¹¹	
	N	%	N	%	N	%
Hematoma	11	3.5	4	2.0	6	3.8
Citrate toxicity	10	3.2	3	1.5	4	2.5
Other	13	4.2	13	6.5	4	2.5

Table 6 summarizes the distribution of adverse reactions versus no adverse reactions across the three target-yield thresholds used during plateletpheresis. The proportions of donors experiencing donor reactions were low and broadly comparable across the three target-yield thresholds. In the 3.0 × 10¹¹ group, 89.1% of donors experienced no adverse reaction, compared with 90.0% in the 4.5 × 10¹¹ group and 91.1% in the 6.0 × 10¹¹ group. Overall, the distribution of adverse reactions across target-yield settings did not differ significantly, indicating that event frequencies remained comparable between groups (p = 0.785).

**Table 6:** Distribution of adverse reactions across target-yield thresholds (N = 670). Note: Comparison of ADR distribution across the three target-yield categories (3.0, 4.5, and 6.0 × 10¹¹ platelets) was performed using the Pearson chi-square test, indicating no statistically significant difference between groups. Statistical significance codes: * p < 0.05, ** p < 0.01, *** p < 0.001.

Adverse reactions	Target yield						
	3 × 10¹¹		4.5 × 10¹¹		6 × 10¹¹		P-value
	N	%	N	%	N	%	
Adverse reactions	34	10.9	20	10	14	8.9	0.785
No adverse reaction	278	89.1	180	90.0	144	91.1	

## Discussion

In this prospective, observational study of 670 plateletpheresis procedures, we demonstrate that platelet yield is primarily determined by intrinsic donor characteristics, most notably pre-donation platelet count, while donor safety, assessed through adverse reaction profiles, is largely preserved across target-yield strategies when contemporary donor selection and apheresis practices are applied. By evaluating yield determinants and donor reactions in parallel within a single cohort, this study provides a clinically relevant synthesis of efficiency and safety considerations that are often examined in isolation.

Our findings confirm pre-donation platelet count as the dominant determinant of platelet yield, exhibiting both the strongest unadjusted correlation and the largest independent effect in multivariable analysis. This observation is consistent with the biological principle that plateletpheresis yield is fundamentally constrained by the circulating platelet pool available for collection. Several prior studies have established baseline platelet count as the most reliable predictor of platelet harvest across apheresis platforms, donor populations, and target yields, reinforcing its central role in donor selection algorithms [5,17-20]. The magnitude of the association observed in our cohort underscores that even in the context of modern automated collection systems, donor platelet reserve remains the principal driver of collection efficiency.

BMI also independently predicted platelet yield, although its contribution was substantially smaller than that of platelet count. Higher BMI likely reflects greater circulating blood volume, permitting larger volumes to be processed while maintaining hemodynamic stability and collection efficiency [17]. This relationship has been reported previously and aligns with operational practices in which donors with larger body habitus are preferentially selected for higher target yields or double-dose collections [19,21,22]. In contrast, age demonstrated only a weak negative association with yield and did not retain independent significance after adjustment, suggesting that age-related differences in platelet kinetics are modest once platelet count and body size are taken into account [5,6,23]. Notably, some studies have reported no independent association between donor body weight or BMI and platelet yield, underscoring that the contribution of body habitus may vary according to donor population, device characteristics, and analytical approach [5,24].

Hematocrit showed a small inverse association with platelet yield, a finding that has been variably reported in earlier studies [25,26]. Higher hematocrit may influence flow dynamics and separation efficiency within the centrifuge chamber by increasing blood viscosity, thereby marginally reducing platelet collection efficiency. Hemoglobin concentration did not independently predict yield, consistent with prior reports indicating that hemoglobin serves primarily as a safety threshold rather than a determinant of platelet harvest once minimum eligibility criteria are met [5,27]. In contrast to our findings, Guerrero-Rivera et al. and El-Enein et al. reported an inverse association between pre-donation hemoglobin and platelet yield, attributing higher yields in donors with lower hemoglobin to increased plasma volume processed during apheresis [18,28].

With respect to donor safety, adverse reactions occurred in approximately 10% of procedures, with the majority being mild and self-limiting. This reaction rate falls within the range reported in Indian and regional plateletpheresis studies and is comparable to those observed in larger international series [8,29]. Hematoma and citrate-related symptoms constituted the most frequent events, while other reactions, such as giddiness and nausea or vomiting, were infrequent. Importantly, no increase in serious or procedure-limiting reactions was observed, reflecting the effectiveness of donor screening, procedural monitoring, and post-donation observation protocols.

In multivariable analysis, BMI emerged as the sole independent donor-level predictor of adverse reactions, with higher BMI conferring a protective effect. This finding is biologically plausible and aligns with donor vigilance literature suggesting that donors with greater body mass and circulating volume are better able to tolerate extracorporeal circulation and anticoagulant exposure [13,15]. The absence of an independent association between age and adverse reactions after adjustment suggests that age-related differences observed in univariate analyses are likely confounded by body habitus and other donor characteristics rather than representing a direct effect of age itself. Notably, pre-donation platelet count was not independently associated with adverse reactions in adjusted analysis, despite its strong influence on yield. This dissociation underscores an important clinical distinction: factors that optimize collection efficiency do not necessarily increase donor risk. While higher platelet counts facilitate yield attainment, they do not appear to predispose donors to increased procedural intolerance within standard operating conditions. This observation supports current donor selection practices that emphasize platelet count as a yield determinant without undue concern for safety trade-offs.

A central finding of this study is the absence of a significant difference in adverse reaction rates across predefined target-yield thresholds. Donor reaction frequencies and patterns were comparable across the 3.0, 4.5, and 6.0 × 10¹¹ platelet targets, with no evidence of a stepwise increase in risk at higher yields. These results are consistent with prior reports indicating that high-yield and double-dose plateletpheresis can be performed safely in appropriately selected donors [8,12]. Although higher target yields may entail longer procedure durations and greater anticoagulant exposure, these potential stressors appear to be effectively mitigated by donor selection based on platelet count and body size, as well as by advances in apheresis technology and real-time monitoring.

The lack of a target-yield effect on donor reactions highlights the importance of distinguishing donor-related susceptibility from procedural strategy. In routine practice, donors are not randomly allocated to target yields; rather, higher yields are preferentially assigned to donors with favorable baseline characteristics. As a result, crude comparisons of reaction rates across yield categories may reflect underlying donor case-mix rather than the intrinsic risk of higher-yield collection. By examining donor characteristics and target-yield groups concurrently, our findings suggest that when donor selection is appropriately applied, escalation to higher yields does not compromise donor safety.

The integrated assessment of platelet yield and donor safety within a single cohort represents a key strength of this study. Many prior investigations have focused either on yield optimization or donor reactions in isolation, limiting their applicability to real-world decision-making where efficiency and safety must be balanced simultaneously. Our findings provide evidence that platelet yield optimization, when guided by donor platelet count and body habitus, can be achieved without increasing adverse reactions, thereby supporting sustainable plateletpheresis practices in high-demand settings.

Several limitations should be acknowledged. This was a single-center study using a single apheresis platform, which may limit generalizability. Adverse reactions were analyzed as a binary outcome in logistic regression due to low event counts, and reaction severity was not further stratified. Additionally, procedures terminated prematurely due to adverse events were excluded, which may underestimate the overall reaction burden. Information on donor experience (first-time donors versus previously experienced donors) was not captured in the analysis. Donor experience is known to influence both platelet yield and the risk of adverse reactions, and its inclusion may further refine predictive models in future studies. Finally, the observational design of the study precludes causal inference. The findings reflect associations observed under routine donor selection practices and should not be interpreted as demonstrating a causal relationship or universal safety of higher target-yield plateletpheresis.

## Conclusions

This study reinforces pre-donation platelet count as the principal determinant of plateletpheresis yield, with BMI also identified as an independent determinant of yield, albeit with a smaller effect size. Donor adverse reactions were infrequent, predominantly mild, and independently associated only with lower BMI. Importantly, higher target-yield plateletpheresis was not associated with an increased risk of donor reactions. In the context of a progressively shrinking platelet donor pool, these findings are particularly relevant, as safely achieving higher yields from eligible donors may help augment platelet inventory and improve component availability for diverse transfusion needs. Overall, the results support integrated donor selection and collection strategies that optimize yield and enhance the efficiency and sustainability of plateletpheresis services in contemporary transfusion practice.
